# Supplementary material for: Tumor exosome-based nanoparticles are efficient drug carriers for chemotherapy
Source: Nat Commun. 2019 Aug 23;10:3838. doi: 10.1038/s41467-019-11718-4 (PMC6707218; doi:10.1038/s41467-019-11718-4)
Supplement: Supplementary file 1 — Supplementary Information [file 41467_2019_11718_MOESM1_ESM.pdf]

Tumor exosome-based nanoparticles are efficient drug carriers for chemotherapy

Yong et al.

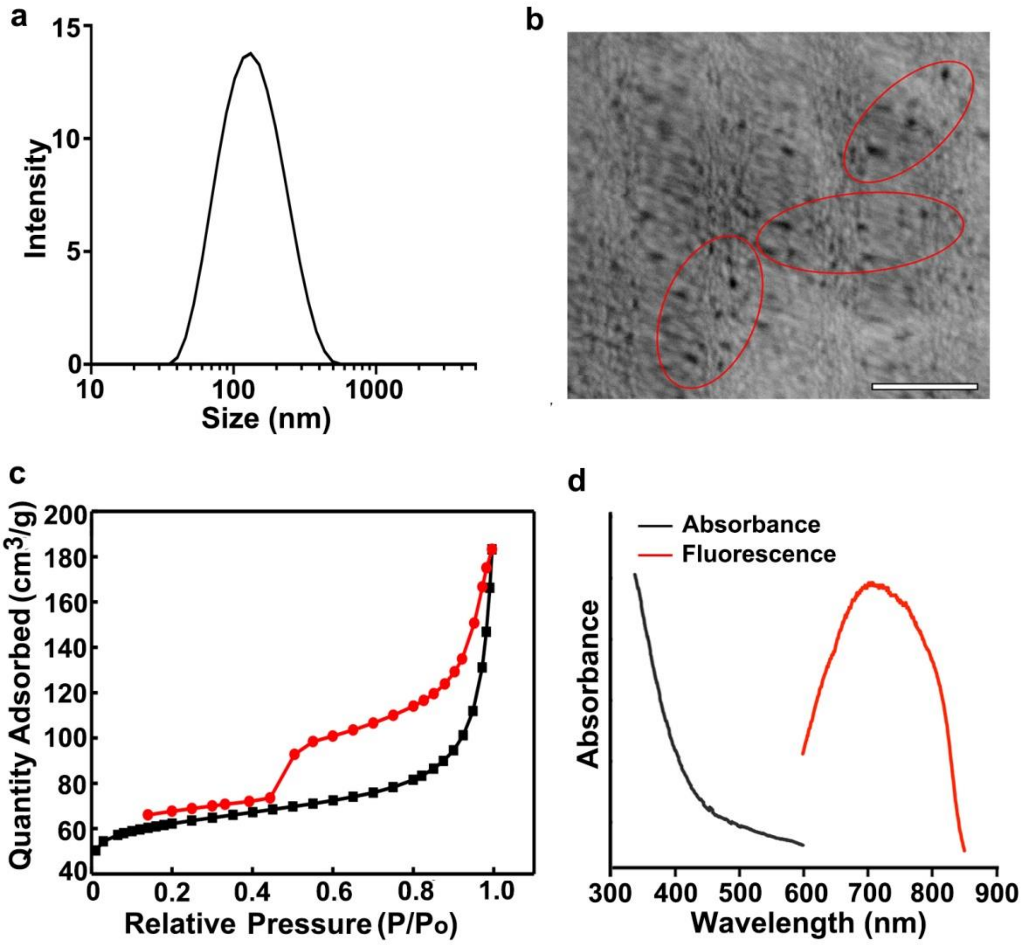

Supplementary Fig. 1. Characterization of PSiNPs. **a** Hydrodynamic diameter of PSiNPs by DLS analysis. **b** SEM image of porous silicon film. Scale bar: 100 nm. **c** N_2 adsorption/desorption isotherms of PSiNPs. **d** Fluorescence emission (Ex.= 488 nm) and absorbance spectra of PSiNPs. Source data are provided as a Source Data file.

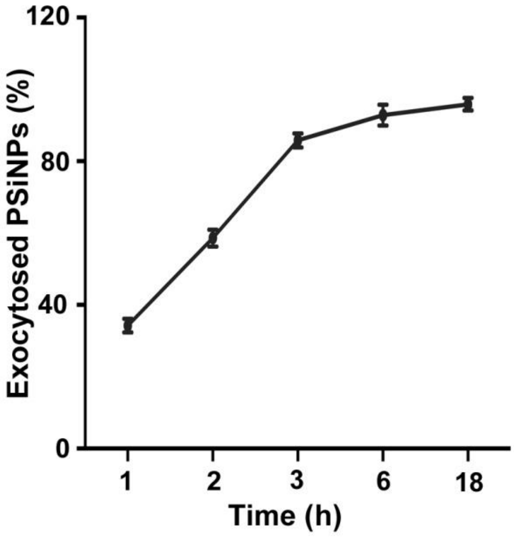

Supplementary Fig. 2. The exocytosis of PSiNPs when Bel7402 cells were pretreated with 200 μg/mL PSiNPs for 6 h and then cultured in fresh medium without PSiNPs for different time points analyzed by ICP-OES. Data were presented as mean ± SD (n =3). Source data are provided as a Source Data file.

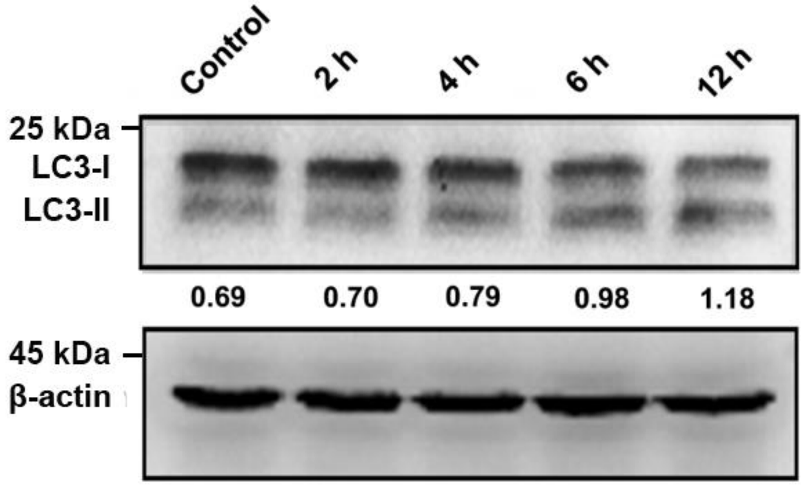

Supplementary Fig. 3. LC3-I and LC3-II expression in H22 cells treated with 200 μg/mL PSiNPs for different time courses by western blot. The number underneath each group in the immunoblotting indicates the relative ratio of LC3-II to LC3-I of the corresponding group. Source data are provided as a Source Data file.

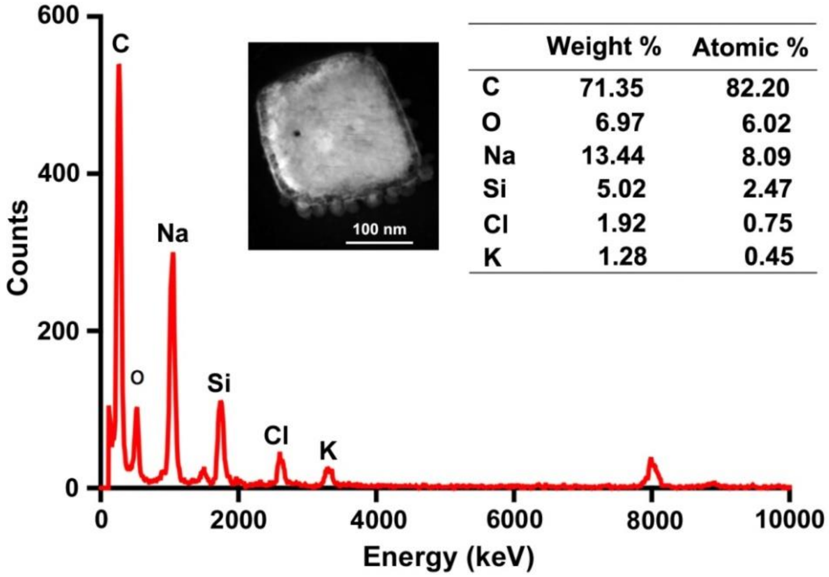

Supplementary Fig. 4. Elemental composition of E-PSiNPs analyzed by FTEM energy spectrum. Source data are provided as a Source Data file.

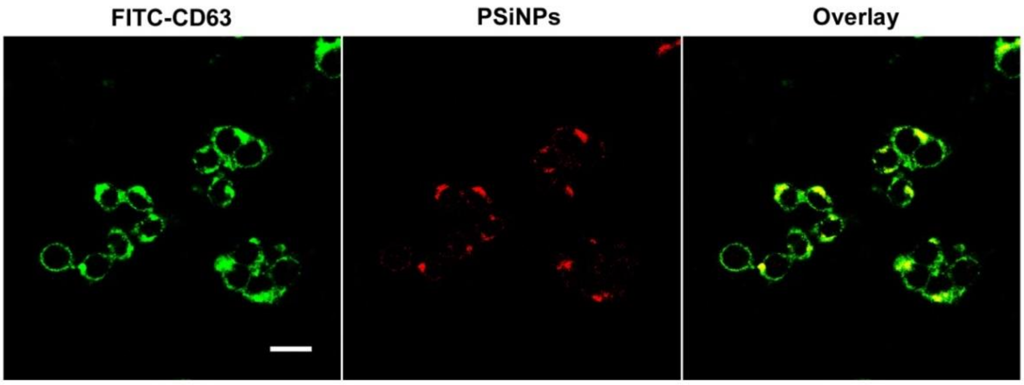

Supplementary Fig. 5. Colocalization of CD63-labelled MVBs (green) and PSiNPs (red) when Bel7402 cells were treated with 75 μg/mL PSiNPs for 2 h analyzed by confocal fluorescence microscopy. Scale bar: 10 μm.

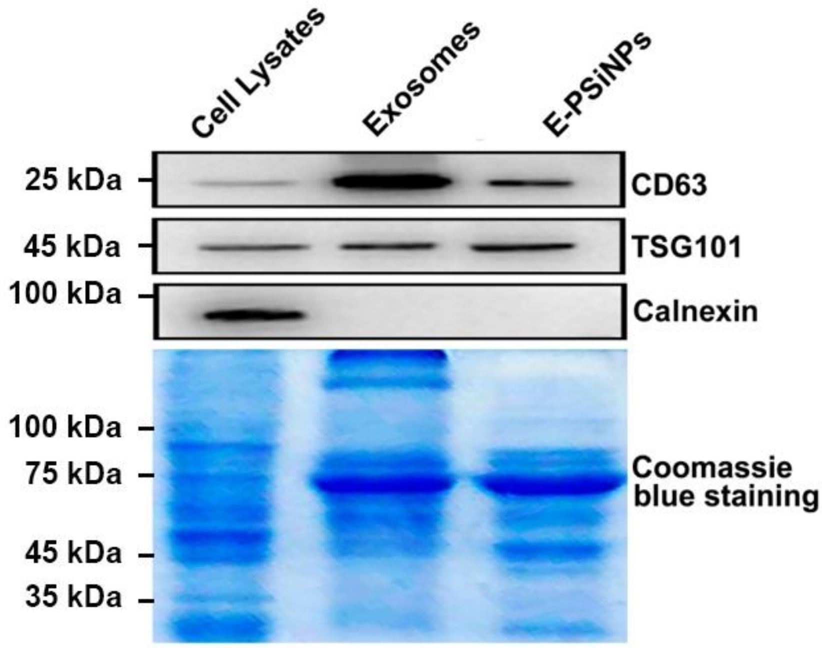

Supplementary Fig. 6. Immunoblotting analysis of exosome markers (TSG101 and CD63) and ER marker (calnexin) expressed in E-PSiNPs exocytosed from H22 cells. Source data are provided as a Source Data file.

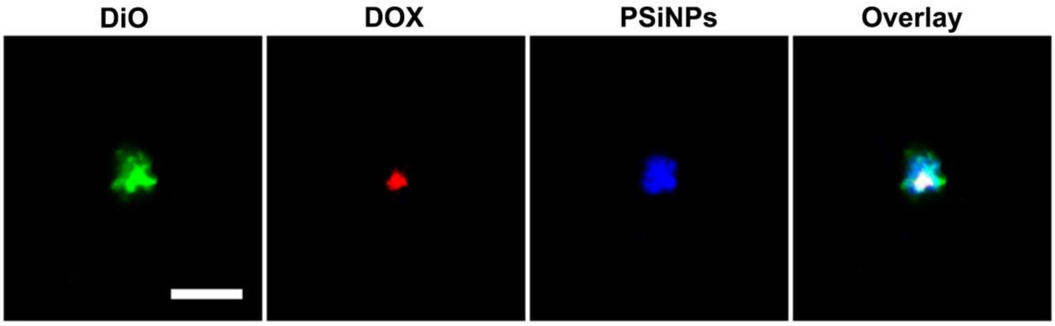

Supplementary Fig. 7. Colocalization of DiO, DOX and PSiNPs in DOX@E-PSiNPs exocytosed from H22 cells by confocal microscopy. Scale bar: 1 μm.

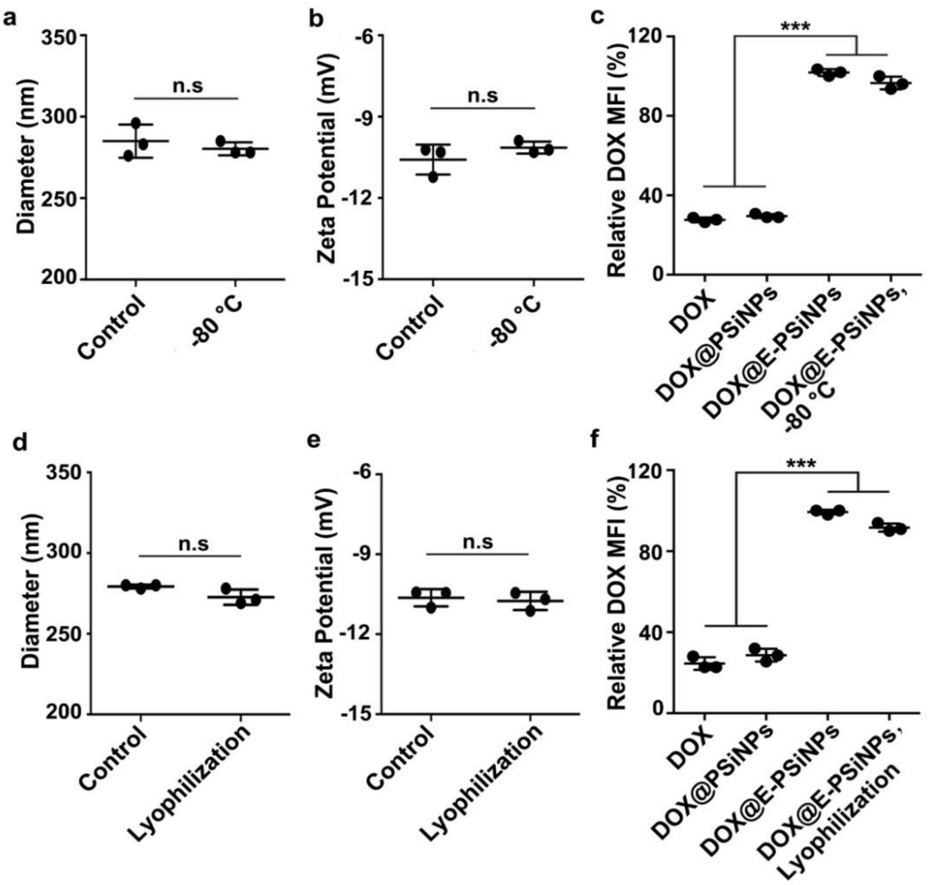

Supplementary Fig. 8. Effects of storage of DOX@E-PSiNPs at −80 ℃ or by lyophilization on their characterization and biological functions. **a, b** Hydrodynamic diameter (**a**) and zeta-potential (**b**) of DOX@E-PSiNPs after storage at −80 ℃ for 1 month. **c, f** Intracellular DOX fluorescence intensity in H22 CSCs after treatment with free DOX, DOX@PSiNPs, DOX@E-PSiNPs or DOX@E-PSiNPs stored at −80 ℃ for 1 month (**c**) or lyophilized followed by resuspension in PBS 1 week later (**f**) at DOX concentration of 2 μg/mL for 2 h by flow cytometry. **d, e** Hydrodynamic diameter (**d**) and zeta potential (**e**) of DOX@E-PSiNPs after lyophylization followed by resuspension in PBS 1 week later. Data were presented as mean ± SD (n=3). ***P<0.001 (unpaired two-tailed Student's t test for **a**, **b**, **d**, **e** and one-way ANOVA with Bonferroni's multiple comparisons test for **c**, **f**). n.s, not statistically significant. Source data are provided as a Source Data file.

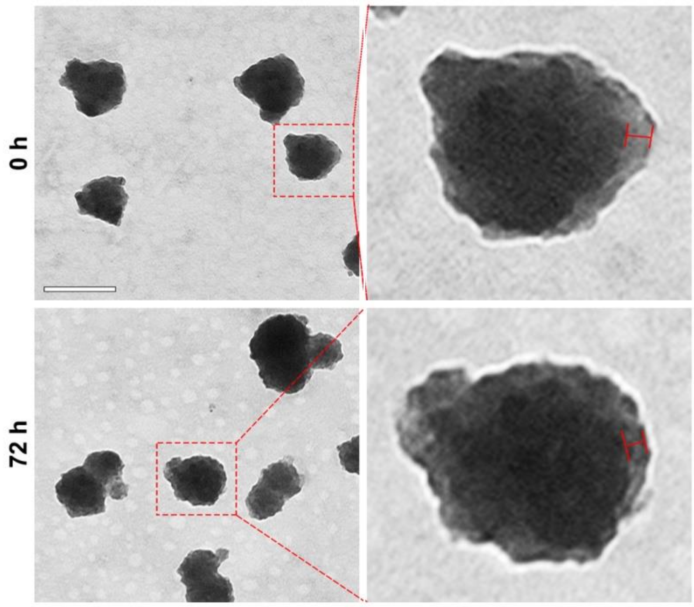

Supplementary Fig. 9. TEM images of DOX@E-PSiNPs after incubation in PBS for 72 h. Scale bar: 200 nm.

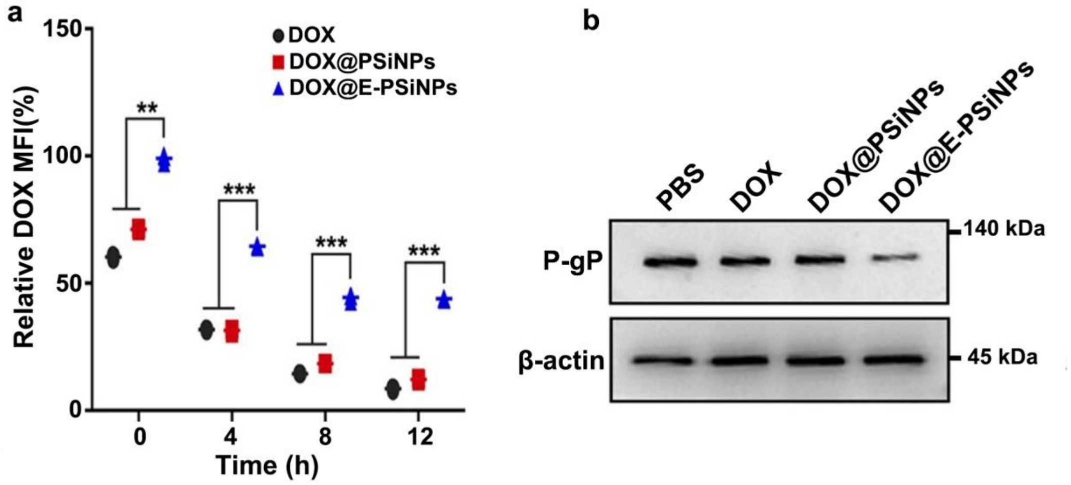

Supplementary Fig. 10. Intracellular retention of DOX@E-PSiNPs in H22 CSCs. **a** Relative DOX mean fluorescence intensity in H22 CSCs after treatment with free DOX, DOX@PSiNPs or DOX@E-PSiNPs exocytosed from H22 cells at DOX concentration of 2 μg/mL for 2 h, followed by washing with PBS and incubating in fresh medium for different time intervals by flow cytometry. Data were presented as mean ± SD (n=3). ***P*<0.01, ****P*<0.001 (two-way ANOVA with Bonferroni's multiple comparisons test). **b** P-gp expression in H22 CSCs after treatment with free DOX, DOX@PSiNPs or DOX@E-PSiNPs exocytosed from H22 cells at DOX concentration of 2 μg/mL for 24 h by western blot. Source data are provided as a Source Data file.

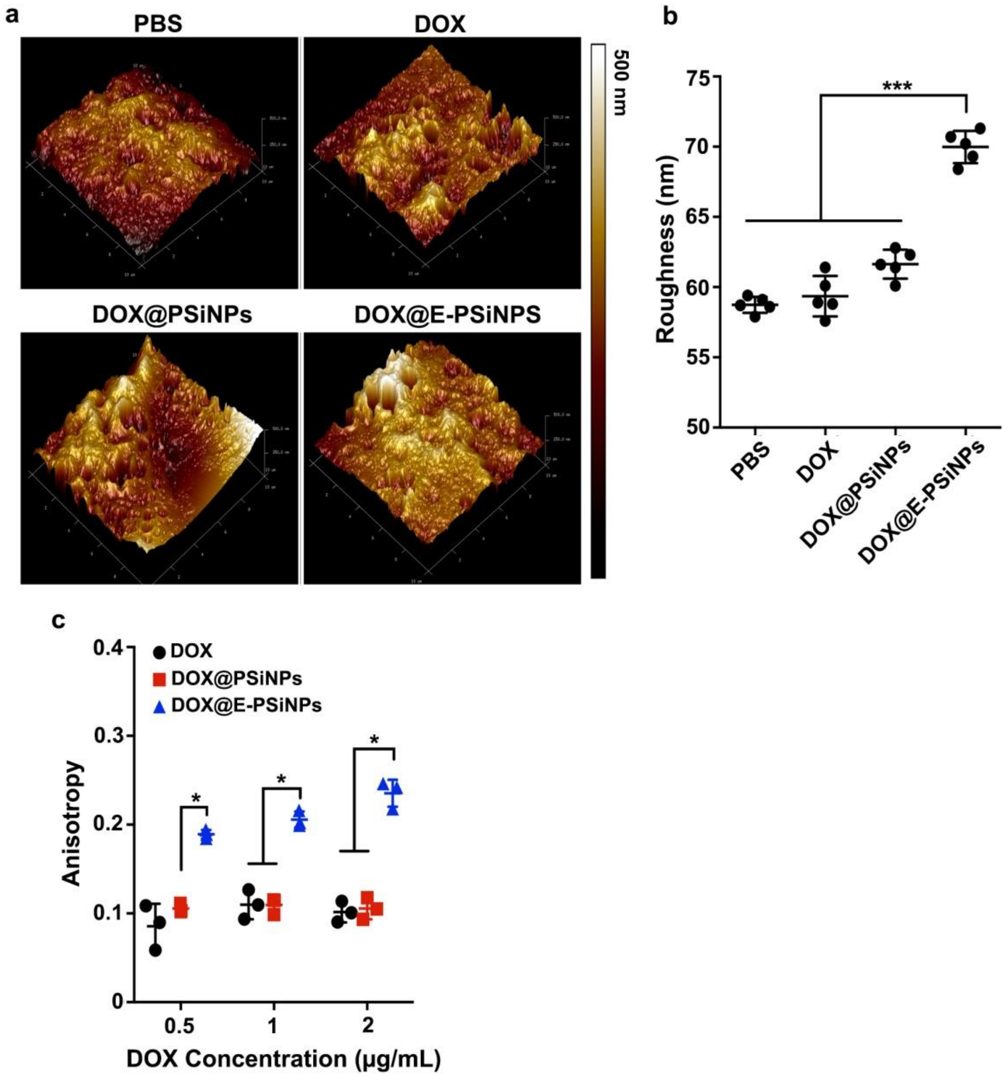

Supplementary Fig. 11. Interaction of DOX@E-PSiNPs with H22 CSCs by AFM. **a** AFM images of H22 CSCs after treatment with DOX, DOX@PSiNPs or DOX@E-PSiNPs at the DOX concentration of 2 μg/mL at 37 ℃ for 2 h. **b** Roughness of H22 CSCs after treatment as above. **c** Cell membrane fluidity of H22 CSCs after treatment with DOX, DOX@PSiNPs or DOX@E-PSiNPs at the DOX concentration of 2 μg/mL for 2 h. Data were presented as mean ± SD (n=3). *P<0.05 (one-way ANOVA with Bonferroni's multiple comparison test for **b** and two-way ANOVA with Bonferroni's multiple comparisons test for **c**). Source data are provided as a Source Data file.

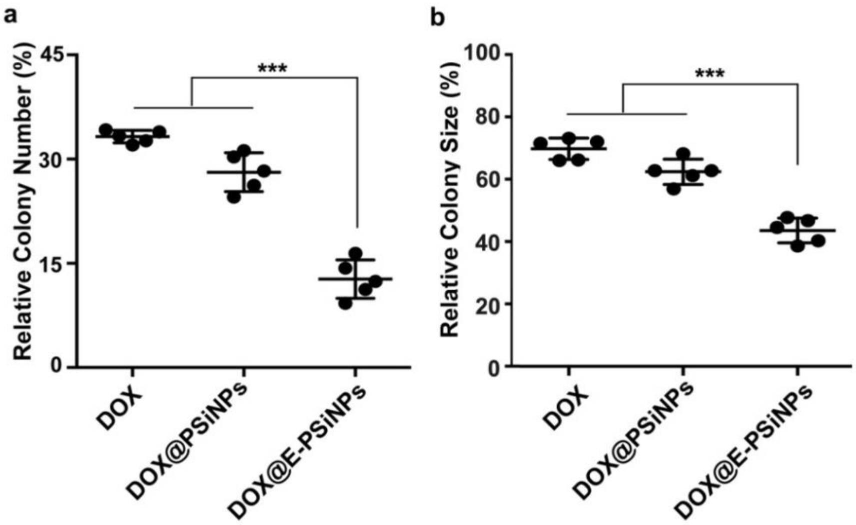

Supplementary Fig. 12. Cytotoxicity of DOX@E-PSiNPs exocytosed from H22 cells against H22 CSCs. **a, b** Relative colony number (**a**) and size (**b**) of tumor spheroids when H22 CSCs selected in soft 3D fibrin gels were treated with free DOX, DOX@PSiNPs or DOX@E-PSiNPs exocytosed from H22 cells at DOX concentration of 2 μg/mL for 24 h. Data were presented as mean ± SD (n=5). ***P<0.001 (one-way ANOVA with Bonferroni's multiple comparison test). Source data are provided as a Source Data file.

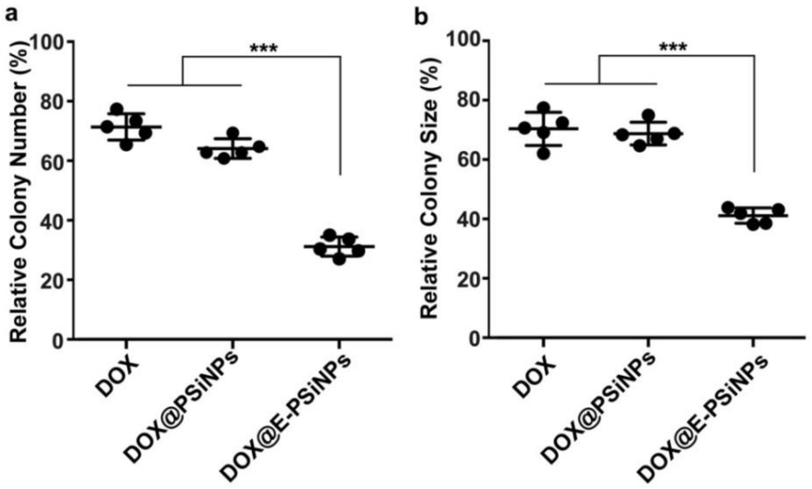

Supplementary Fig. 13. Cytotoxicity of DOX@E-PSiNPs exocytosed from H22 cells against B16-F10 CSCs. **a, b** Relative colony number (**a**) and size (**b**) of tumor spheroids when B16-F10 CSCs selected in soft 3D fibrin gels were treated with free DOX, DOX@PSiNPs or DOX@E-PSiNPs exocytosed from H22 cells at DOX concentration of 2 μg/mL for 24 h. Data were presented as mean ± SD (n=5). ***P<0.001 (one-way ANOVA with Bonferroni's multiple comparison test). Source data are provided as a Source Data file.

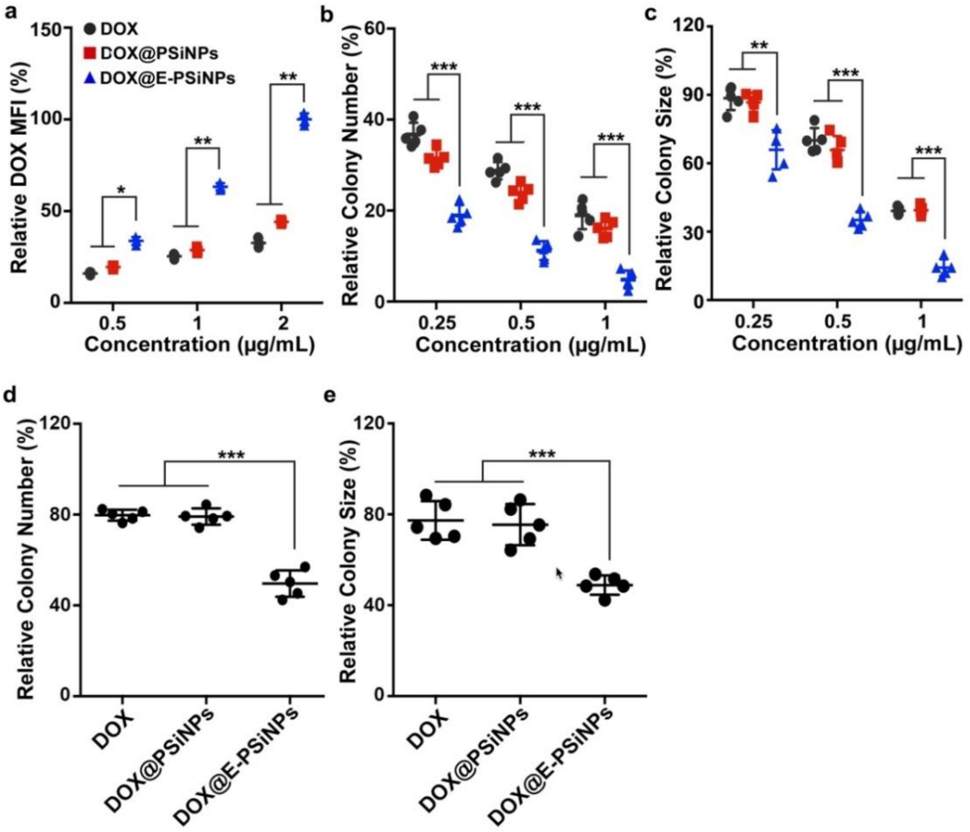

Supplementary Fig. 14. Cross-reactive cellular uptake and cytotoxicity of DOX@E-PSiNPs exocytosed from B16-F10 against H22 CSCs. **a** Relative DOX fluorescence intensity when H22 CSCs selected in soft 3D fibrin gels were treated with free DOX, DOX@PSiNPs or DOX@E-PSiNPs exocytosed from B16-F10 cells at different DOX concentrations for 2 h by flow cytometry. Data were represented as mean ± SD (n=3). **b, c** Relative colony number (**b**) and size (**c**) of tumor spheroids when H22 CSCs were pretreated with free DOX, DOX@PSiNPs or DOX@E-PSiNPs exocytosed from B16-F10 cells at different DOX concentrations for 4 h and then seeded in soft 3D fibrin gels for 5 days. **d, e** Relative colony number (**d**) and size (**e**) of tumor spheroids when H22 CSCs selected in soft 3D fibrin gels were treated with free DOX, DOX@PSiNPs or DOX@E-PSiNPs exocytosed from B16-F10 cells at DOX concentration of 2 μg/mL for 24 h. Data were presented as mean ± SD (n=5). **P*<0.05, ***P*<0.01, ****P*<0.001 (two-way ANOVA with Bonferroni's multiple comparison test for **a**, **b**, **c** and one-way ANOVA with Bonferroni's multiple comparison test for **d**, **e**). Source data are provided as a Source Data file.

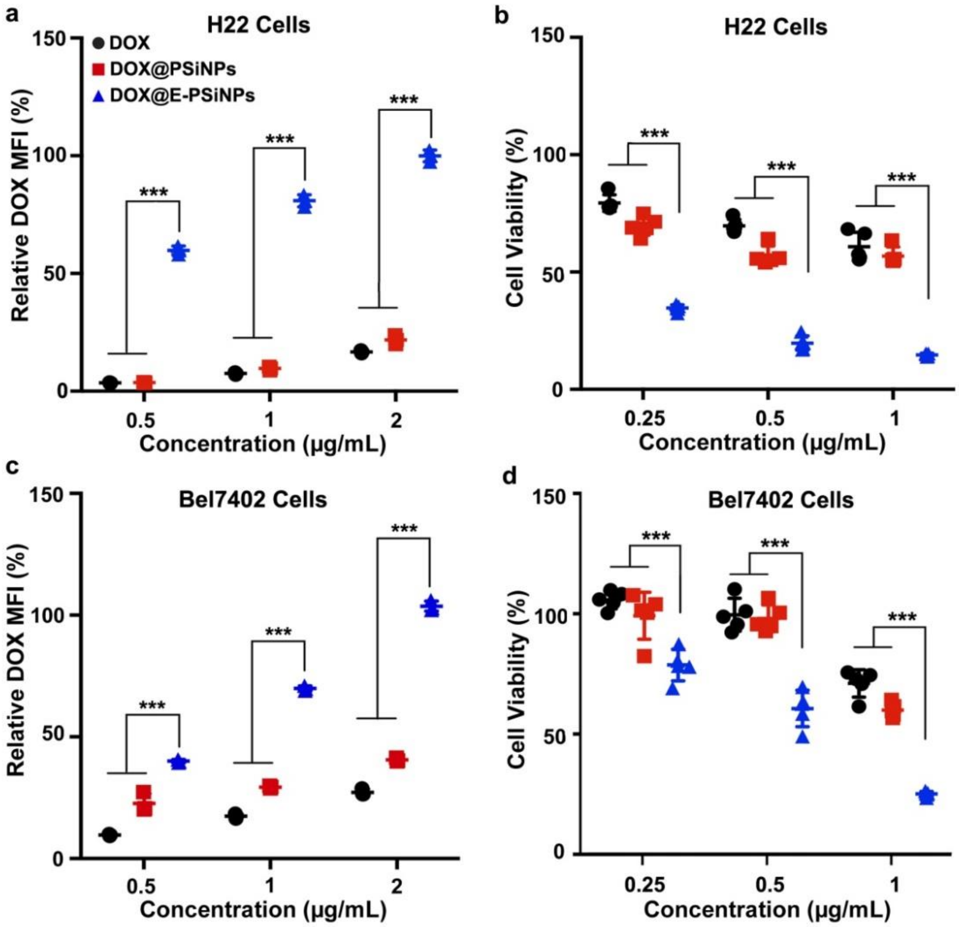

Supplementary Fig. 15. Cellular uptake and cytotoxicity of DOX@E-PSiNPs against bulk cancer cells. **a, c** Relative DOX fluorescence intensity when H22 (**a**) and Bel7402 cells (**c**) were treated with free DOX, DOX@PSiNPs or DOX@E-PSiNPs exocytosed from H22 and Bel7402 cells at different DOX concentrations for 2 h by flow cytometry, respectively. Data were presented as mean ± SD (n=3). **b, d** Cell viability of H22 (**b**) and Bel7402 cells (**d**) treated with free DOX, DOX@PSiNPs or DOX@E-PSiNPs exocytosed from H22 and Bel7402 cells at different DOX concentrations for 24 h by CCK-8 assay, respectively. Data were presented as mean ± SD (n=5). ***P<0.001 (two-way ANOVA with Bonferroni's multiple comparison test). Source data are provided as a Source Data file.

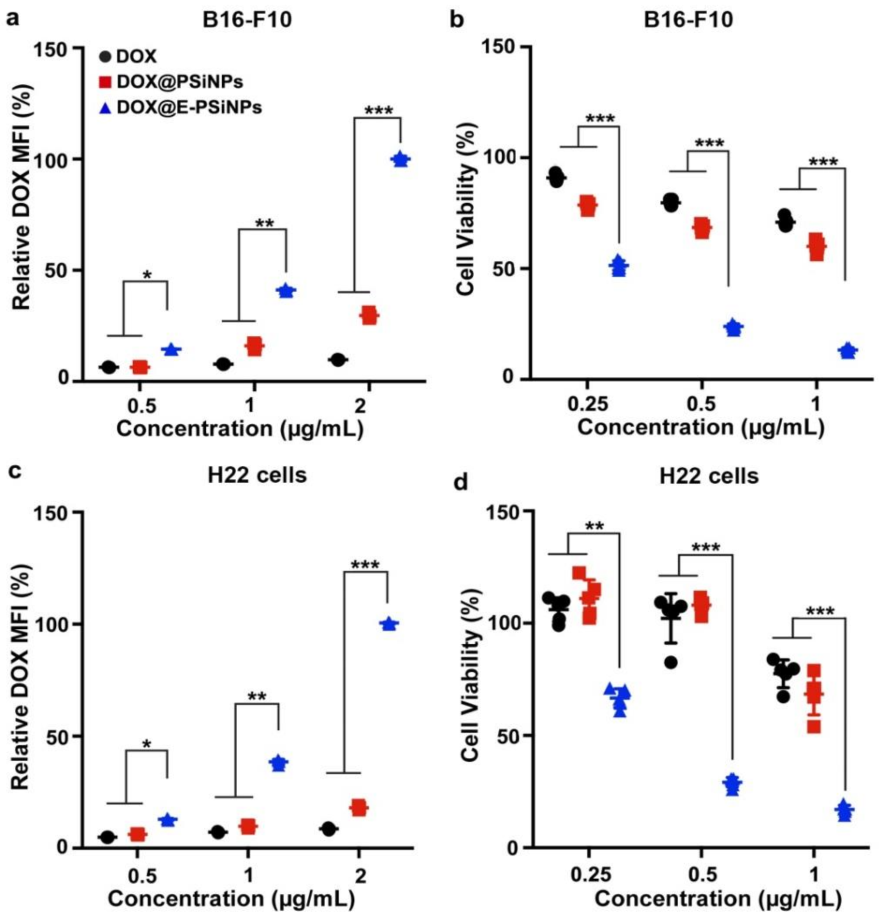

Supplementary Fig. 16. Cross-reactive cellular uptake and cytotoxicity of DOX@E-PSiNPs against bulk cancer cells. **a, c** Relative DOX fluorescence intensity when B16-F10 (**a**) and H22 cells (**c**) were treated with free DOX, DOX@PSiNPs or DOX@E-PSiNPs exocytosed from H22 and B16-F10 cells at different DOX concentrations for 2 h by flow cytometry, respectively. Data were represented as mean ± SD (n=3). **b, d** Cell viability of B16-F10 (**b**) and H22 cells (**d**) treated with free DOX, DOX@PSiNPs or DOX@E-PSiNPs exocytosed from H22 and B16-F10 cells at different DOX concentrations for 24 h by CCK-8 assay, respectively. Data were presented as mean ± SD (n=5). *P<0.05, **P<0.01, ***P<0.001 (two-way ANOVA with Bonferroni's multiple comparison test). Source data are provided as a Source Data file.

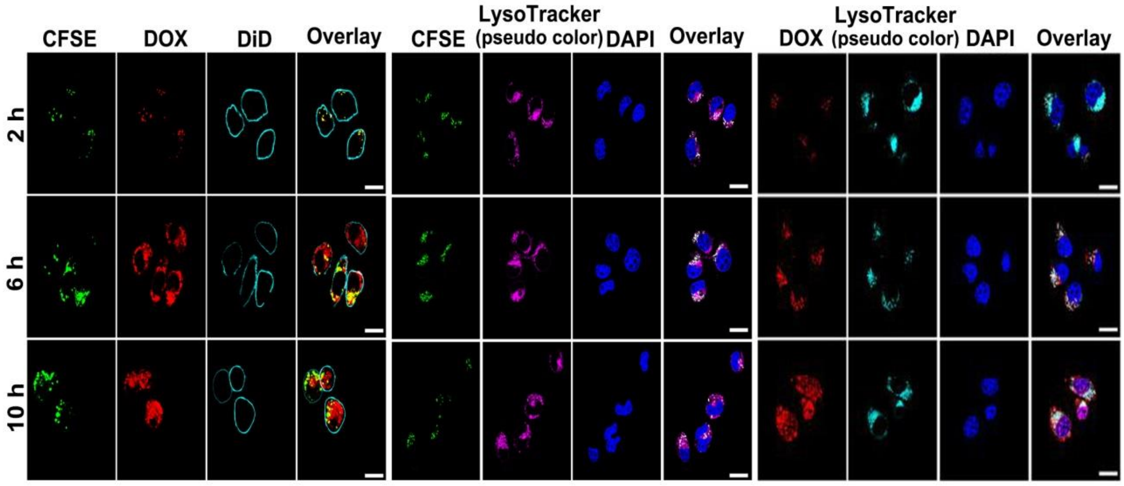

Supplementary Fig. 17. Confocal microscopic images of the intracellular trafficking of CFSE-labeled DOX@E-PSiNPs after B16-F10 cells were treated with DOX@E-PSiNPs at DOX concentration of 2 μg/mL for different time intervals and then labeled with 1 μg/mL DiD (cell membrane labeling dye), 50 nM LysoTracker® Deep Red (lysosomes labeling dye) or 1 μg/mL DAPI (nucleus labeling dye), respectively. Scale bar: 25 μm.

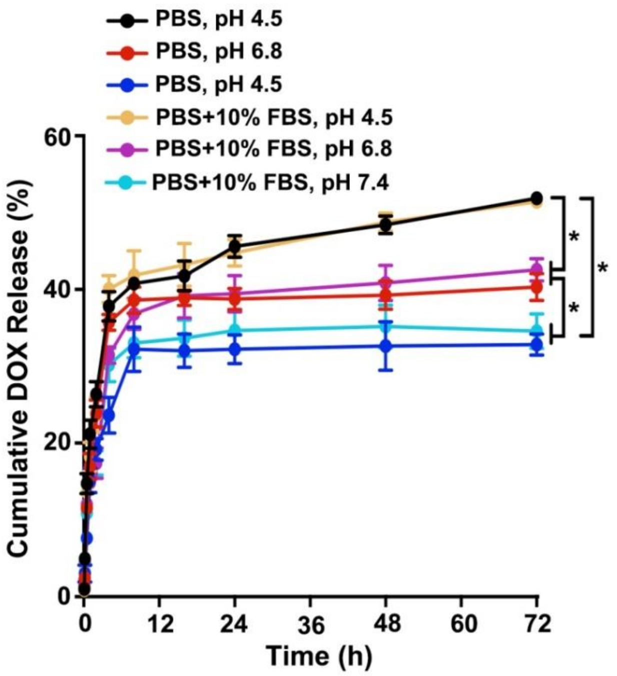

Supplementary Fig. 18. *In vitro* DOX release profiles of DOX@E-PSiNPs in PBS with or without 10% FBS at different pH values by dialysis bag. Data were presented as mean ± SD (n=3). **P*<0.05 (one-way ANOVA with Bonferroni's multiple comparison test). Source data are provided as a Source Data file.

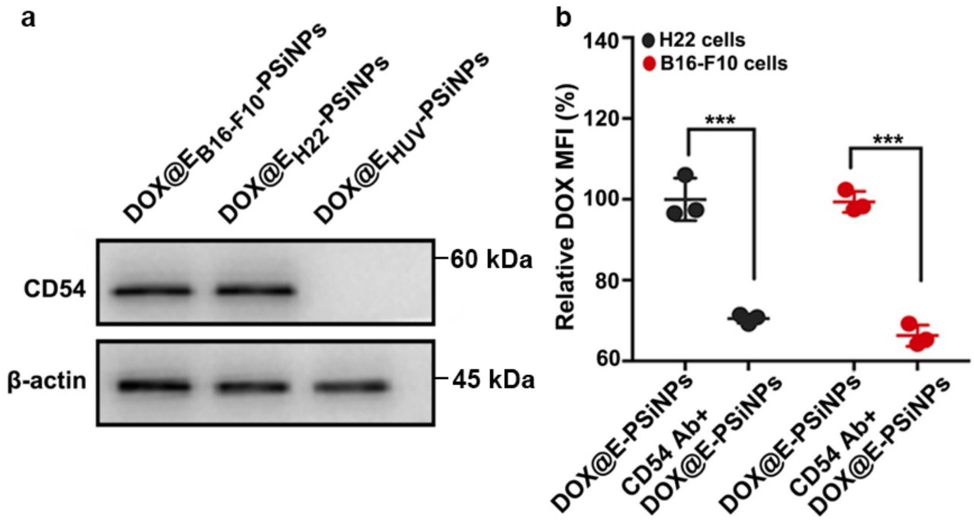

Supplementary Fig. 19. Involvement of CD54 in the cross-reactive cellular uptake of DOX@E-PSiNPs by cancer cells. **a** CD54 expression in DOX@E-PSiNPs exocytosed from B16-F10, H22 or HUVEC cells. **b** Relative DOX fluorescence intensity in H22 and B16-F10 cells at 2 h after treatment with DOX@E-PSiNPs exocytosed from H22 cells pretreated with or without CD54 antibody. Data were represented as mean ± SD (n=3). ****P*<0.001 (unpaired two-tailed Student's *t* test). Source data are provided as a Source Data file.

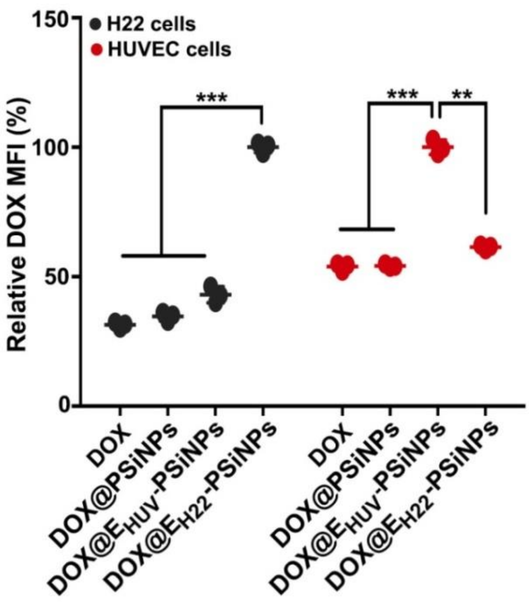

Supplementary Fig. 20. Relative DOX fluorescence intensity in H22 and HUVEC cells after treatment with free DOX, DOX@PSiNPs, DOX@E-PSiNPs exocytosed from HUVEC cells or H22 cells at DOX concentration of 2 μg/mL for 2 h, respectively. Data were represented as mean ± SD (n=3). $^{**}P<0.01$, $^{***}P<0.001$ (one-way ANOVA with Bonferroni's multiple comparison test). Source data are provided as a Source Data file.

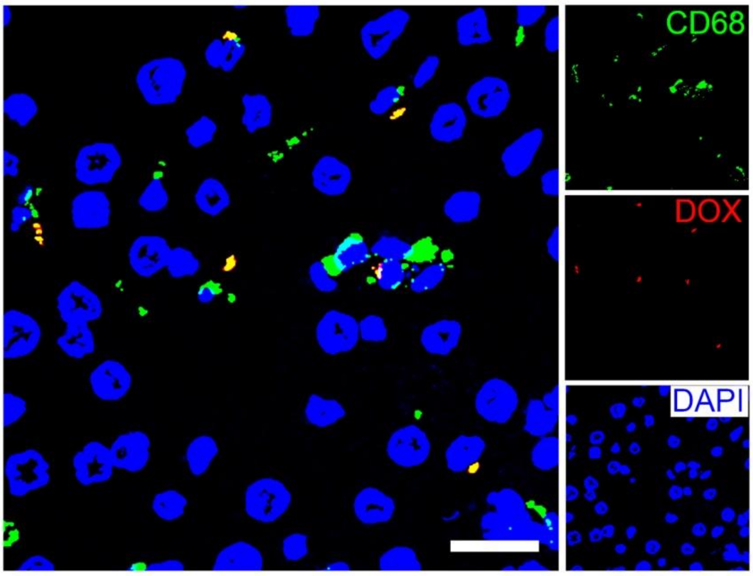

Supplementary Fig. 21. Colocalization of DOX with CD68-labeled Kupffer cells in liver tissues of H22 tumor-bearing mice at 24 h after intravenous injection of DOX@E-PSiNPs at DOX dosage of 0.5 mg/kg. Scale bar: 50 μm.

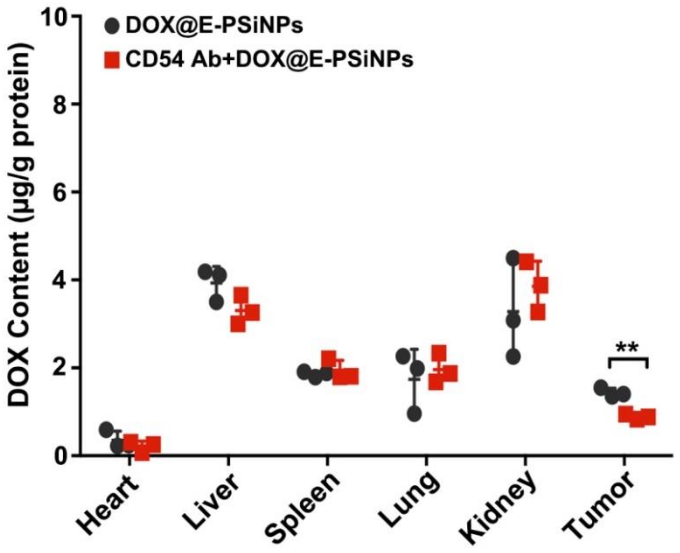

Supplementary Fig. 22. DOX content in tumor tissues and major organs of H22 tumor-bearing mice at 24 h after intravenous injection of DOX@E-PSiNPs exocytosed from H22 cells pretreated with or without CD54 antibody at the DOX dosage of 0.5 mg/kg. Data were represented as mean ± SD (n=3). ***P*<0.01 (unpaired two-tailed Student's *t* test). Source data are provided as a Source Data file.

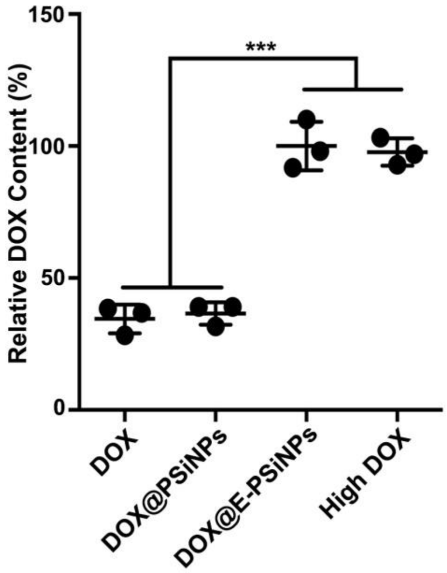

Supplementary Fig. 23. DOX content in lung metastatic nodules of B16-F10 tumor-bearing mice at 24 h after intravenous injection of free DOX, DOX@PSiNPs or DOX@E-PSiNPs at 0.5 mg/kg DOX dosage, or free DOX at 4 mg/kg dosage. Data were represented as mean ± SD (n=3). ***P<0.001 (one-way ANOVA with Bonferroni's multiple comparison test). Source data are provided as a Source Data file.

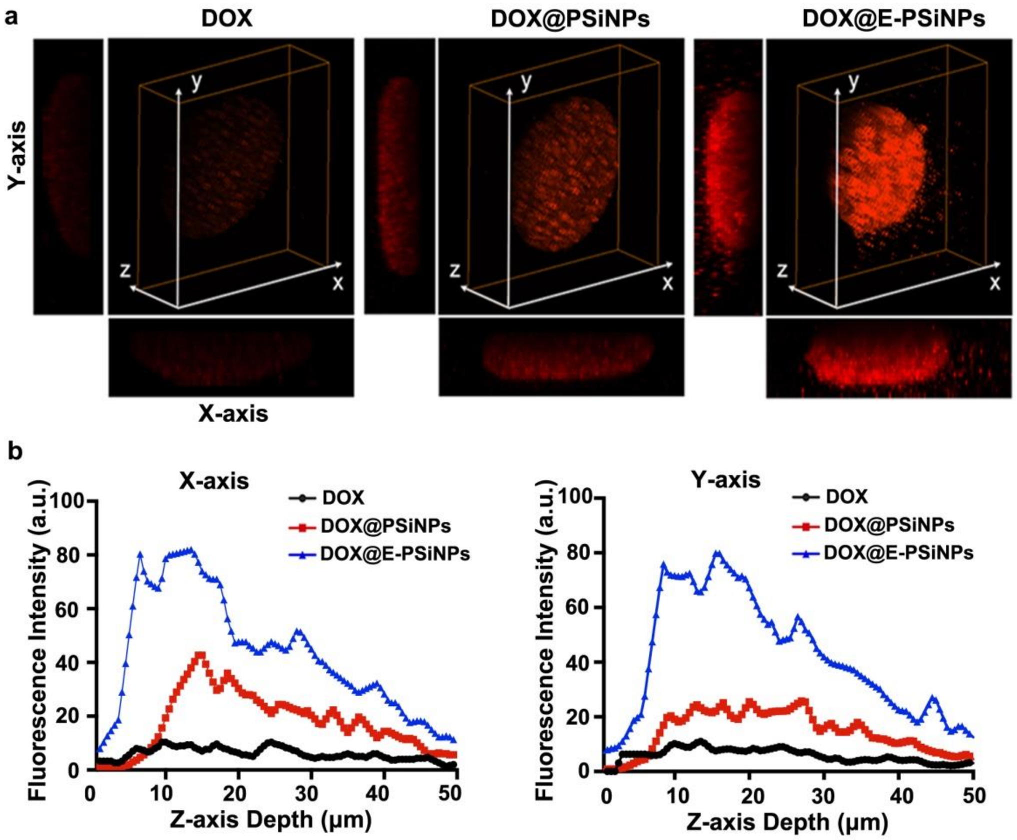

Supplementary Fig. 24. *In vitro* penetration of DOX@E-PSiNPs into tumor spheroids. **a** 3D DOX fluorescence in H22 tumor spheroids after treatment with free DOX, DOX@PSiNPs or DOX@E-PSiNPs at DOX concentration of 2 μg/mL for 24 h, respectively. **b** Relative DOX fluorescence in X- and Y-axis shadows of H22 tumor spheroids. Data were presented as mean ± SD (n=3). Source data are provided as a Source Data file.

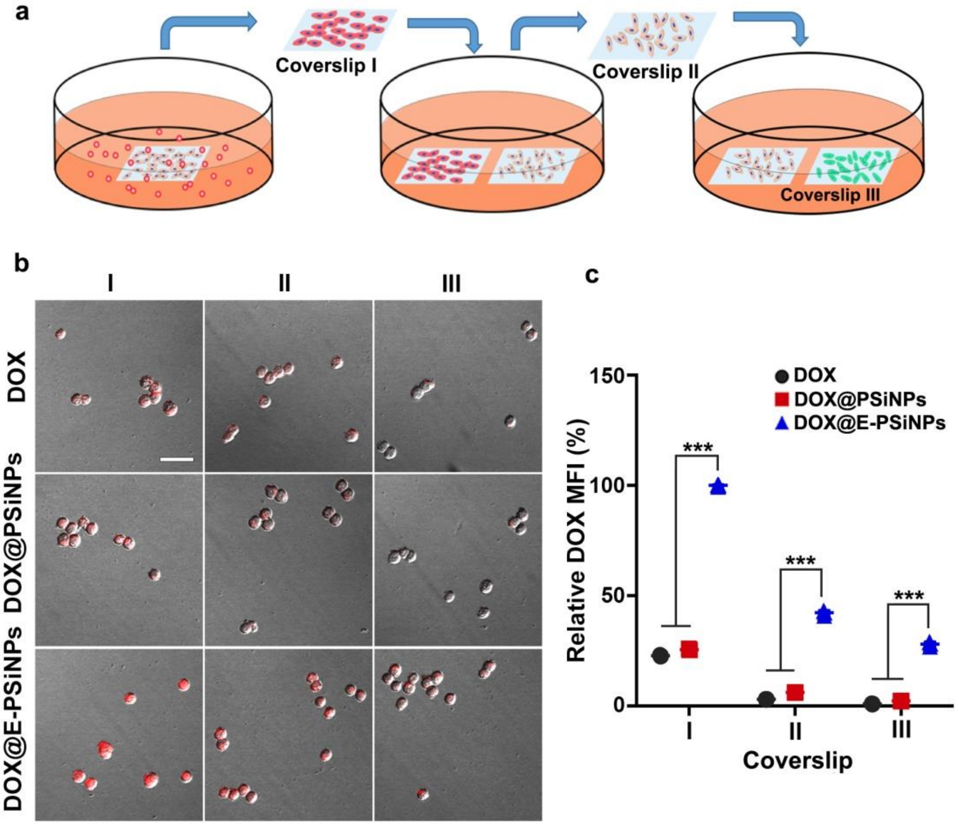

Supplementary Fig. 25. Intercellular delivery of DOX@E-PSiNPs in H22 cells. **a** Diagram of intercellular delivery of DOX@E-PSiNPs. **b** Confocal microscopic images of the successive transport of free DOX, DOX@PSiNPs or DOX@E-PSiNPs from the infected H22 cells to the untreated cells. Scale bar was 50 μm. **c** Relative DOX fluorescence in the successively infected H22 cells by flow cytometry. Data were represented as mean ± SD (n = 3). ***P<0.001 (two-way ANOVA with Bonferroni's multiple comparison test). Source data are provided as a Source Data file.

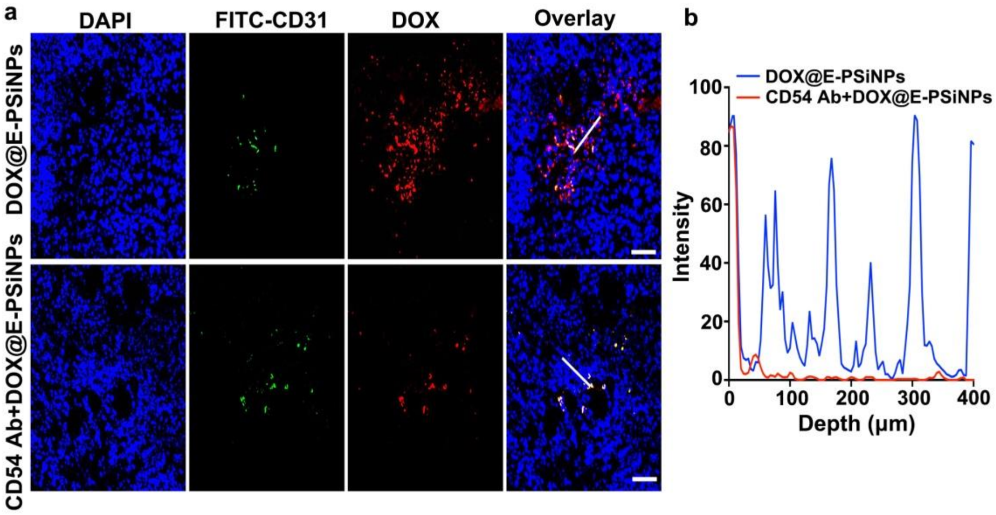

Supplementary Fig. 26. Involvement of CD54 in the tumor penetration of DOX@E-PSiNPs. **a** Colocalization of DOX and CD31-labeled tumor vessels in tumor sections of H22 tumor-bearing mice at 24 h after intravenous injection of DOX@E-PSiNPs pretreated with or without CD54 antibody at DOX dosage of 0.5 mg/kg. Scale bar: 250 μm. White lines represent the distance between DOX in blood vessels and DOX in tumor parenchyma. **b** DOX distribution profile from the blood vessels to tumor parenchyma on the specified white lines as indicated in A. Source data are provided as a Source Data file.

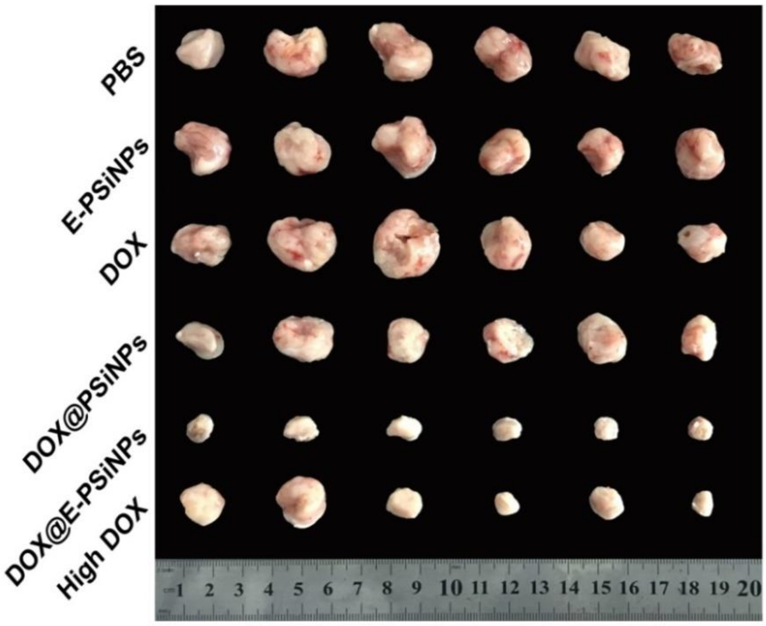

Supplementary Fig. 27. Images of tumor tissues of H22 tumor-bearing mice after intravenous injection of PBS, E-PSiNPs, free DOX, DOX@PSiNPs or DOX@E-PSiNPs at DOX dosage of 0.5 mg/kg, or free DOX at 4 mg/kg dosage once every three day for 5 times.

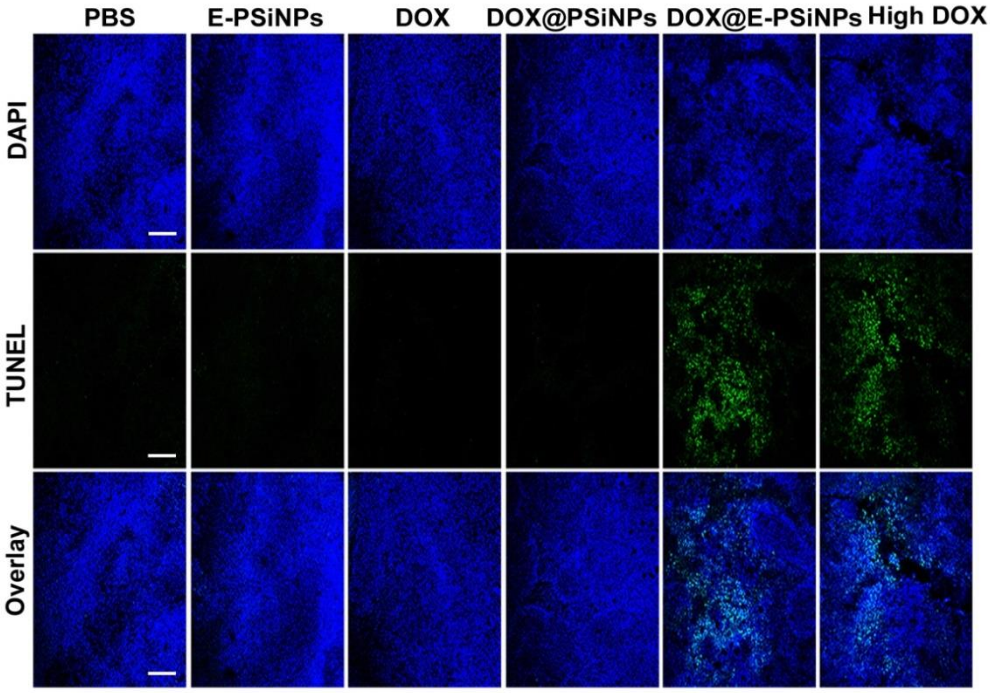

Supplementary Fig. 28. Representative images of TUNEL staining assay in tumor tissues of H22 tumor-bearing mice after intravenous injection of PBS, E-PSiNPs, free DOX, DOX@PSiNPs or DOX@E-PSiNPs at DOX dosage of 0.5 mg/kg, or free DOX at 4 mg/kg dosage once every three day for 5 times. Scale bar: 200 μm.

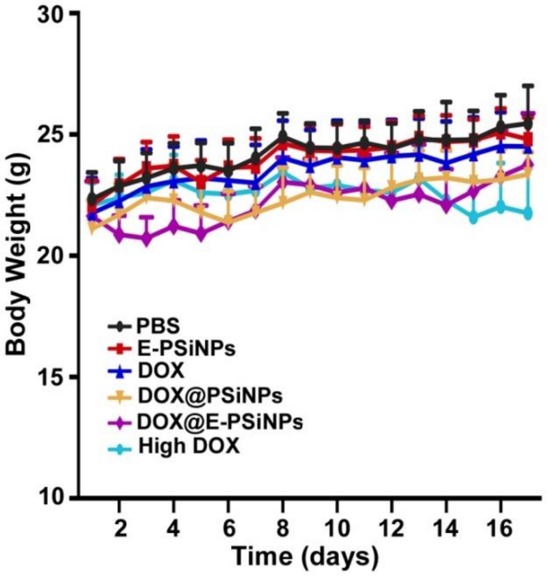

Supplementary Fig. 29. Body weight curves of H22 tumor-bearing mice after intravenous injection of PBS, E-PSiNPs, free DOX, DOX@PSiNPs or DOX@E-PSiNPs at DOX dosage of 0.5 mg/kg, or free DOX at 4 mg/kg dosage once every three day for 5 times. Data were represented as mean ± SD (n=8). Source data are provided as a Source Data file.

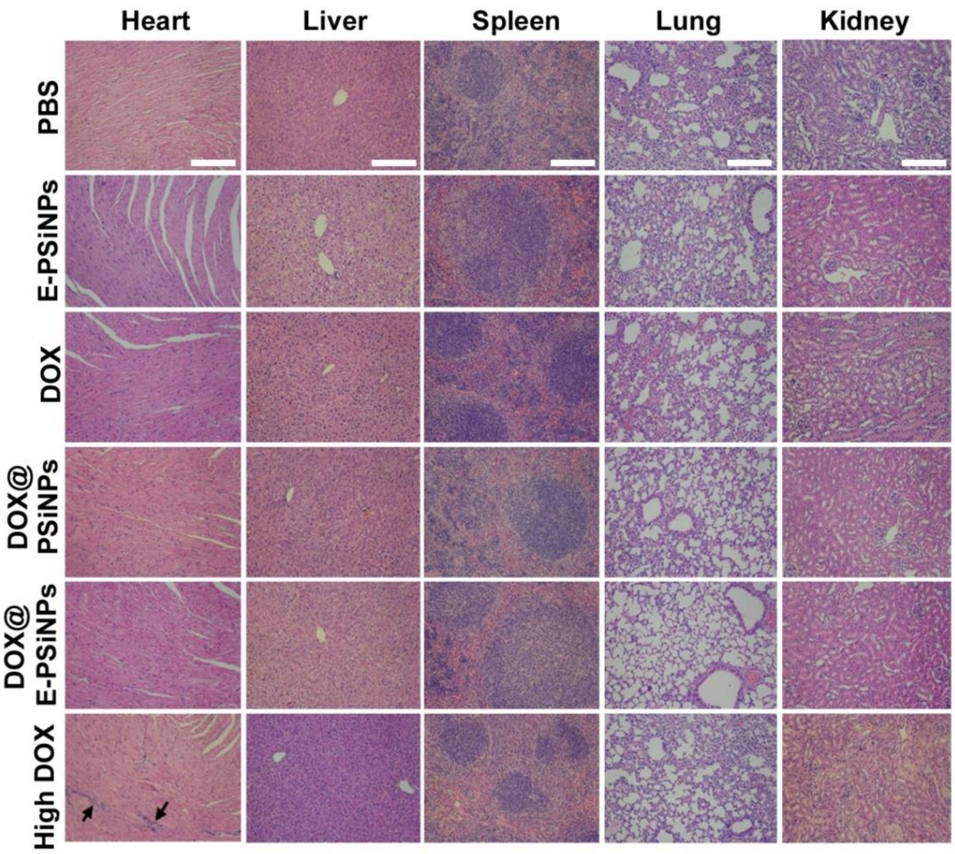

Supplementary Fig. 30. H&E staining of major organs of H22 tumor-bearing mice after intravenous injection of PBS, E-PSiNPs, free DOX, DOX@PSiNPs or DOX@E-PSiNPs at DOX dosage of 0.5 mg/kg, or free DOX at 4 mg/kg dosage once every three day for 5 times. Black arrows represent neutrophil accumulation. Scale bar: 100 μm.

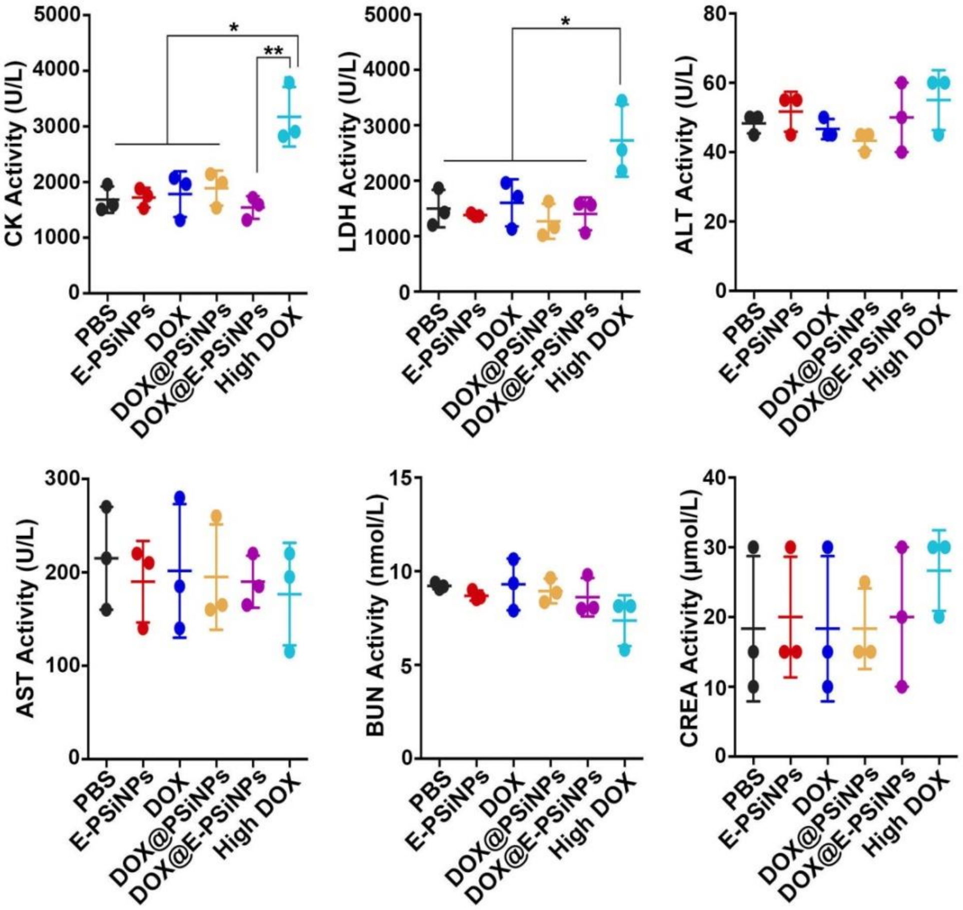

Supplementary Fig. 31. Serological analysis of creatine kinase (CK), lactate dehydrogenase (LDH), alanine aminotransferase (ALT), aspartate aminotransferase (AST), blood urea nitrogen (BUN) and creatinine (CREA) after H22 tumor-bearing mice were intravenously injected with PBS, E-PSiNPs, free DOX, DOX@PSiNPs or DOX@E-PSiNPs at DOX dosage of 0.5 mg/kg, or free DOX at 4 mg/kg dosage once every three day for 5 times. Data were represented as mean ± SD (n=3). **P*<0.05, ***P*<0.01 (one-way ANOVA with Bonferroni's multiple comparison test). Source data are provided as a Source Data file.

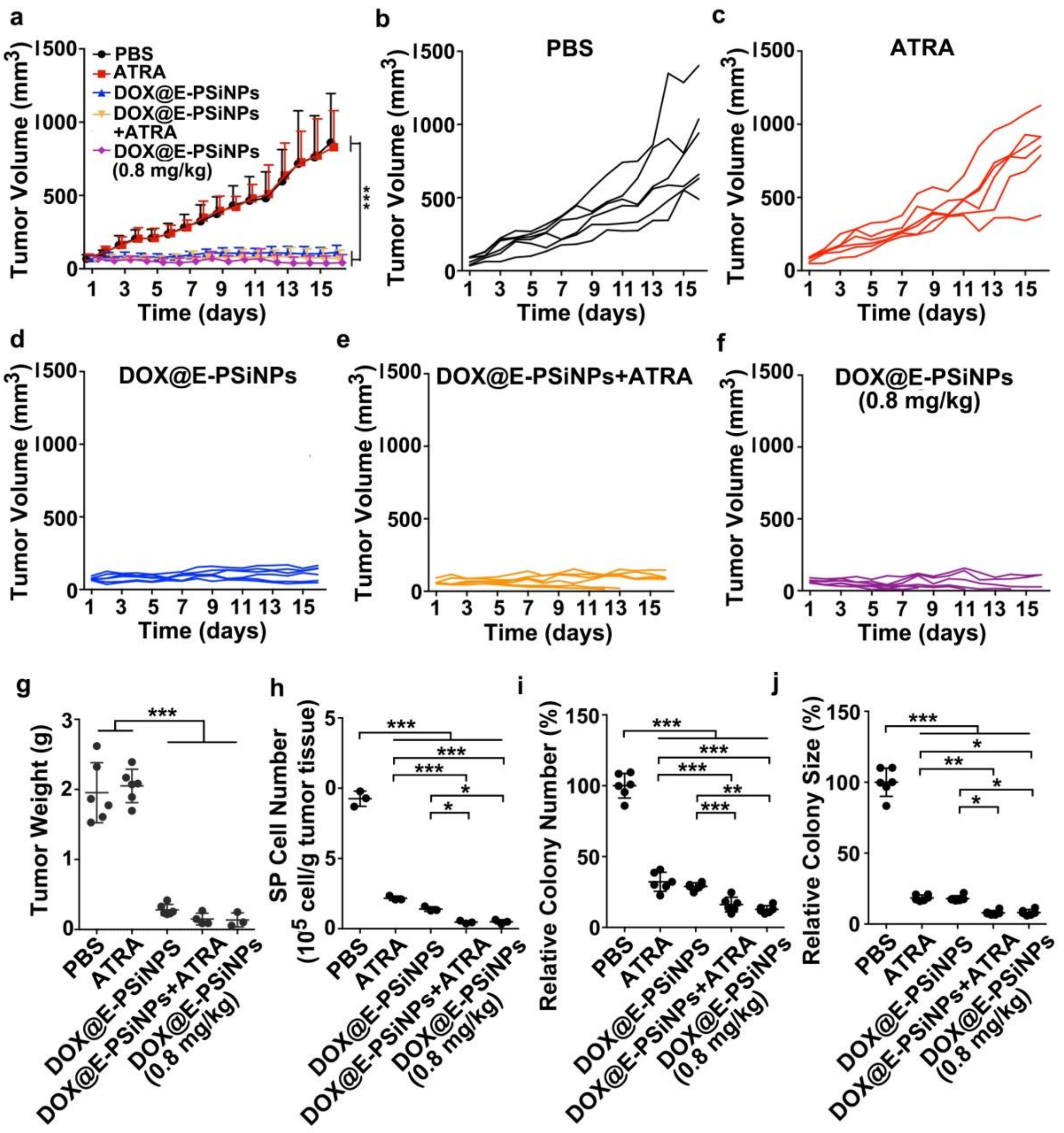

Supplementary Fig. 32. Anticancer and CSCs killing activity of co-injection of DOX@E-PSiNPs and ATRA. **a** Tumor growth curves of H22 tumor-bearing mice after intravenous injection of PBS, ATRA, DOX@E-PSiNPs exocytosed from H22 cells or combination of DOX@E-PSiNPs and ATRA at ATRA and DOX dosage of 4 mg/kg and 0.5 mg/kg, respectively, or DOX@E-PSiNPs at DOX dosage of 0.8 mg/kg every three days for five times. Data were represented as mean ± SD (n=6). **b-f** Tumor growth curves of individual H22 tumor-bearing mice after treatment with PBS (**b**), ATRA (**c**), DOX@E-PSiNPs exocytosed from H22 cells (**d**) or combination of

DOX@E-PSiNPs and ATRA at ATRA and DOX dosage of 4 mg/kg and 0.5 mg/kg (**e**), respectively, or DOX@E-PSiNPs at DOX dosage of 0.8 mg/kg (**f**) as above (n=6). **g** Weight of tumor tissues at the end of tumor growth inhibition experiments. Data were represented as mean ± SD (n=6). **h** Number of side population cells in GFP-positive tumor cells of GFP-expressing H22 tumor-bearing mice at the end of tumor growth inhibition experiments as above. Data were represented as mean ± SD (n=3). **i, j** Relative colony number (**i**) and size (**j**) of tumor spheroids when tumor cells digested from tumor tissues of H22 tumor-bearing mice at the end of tumor growth inhibition experiments were seeded in soft 3D fibrin gels for 5 days. Data were represented as mean ± SD (n=6). *$P<0.05$, **$P<0.01$, ***$P<0.001$ (one-way ANOVA with Bonferroni's multiple comparison test). Source data are provided as a Source Data file.

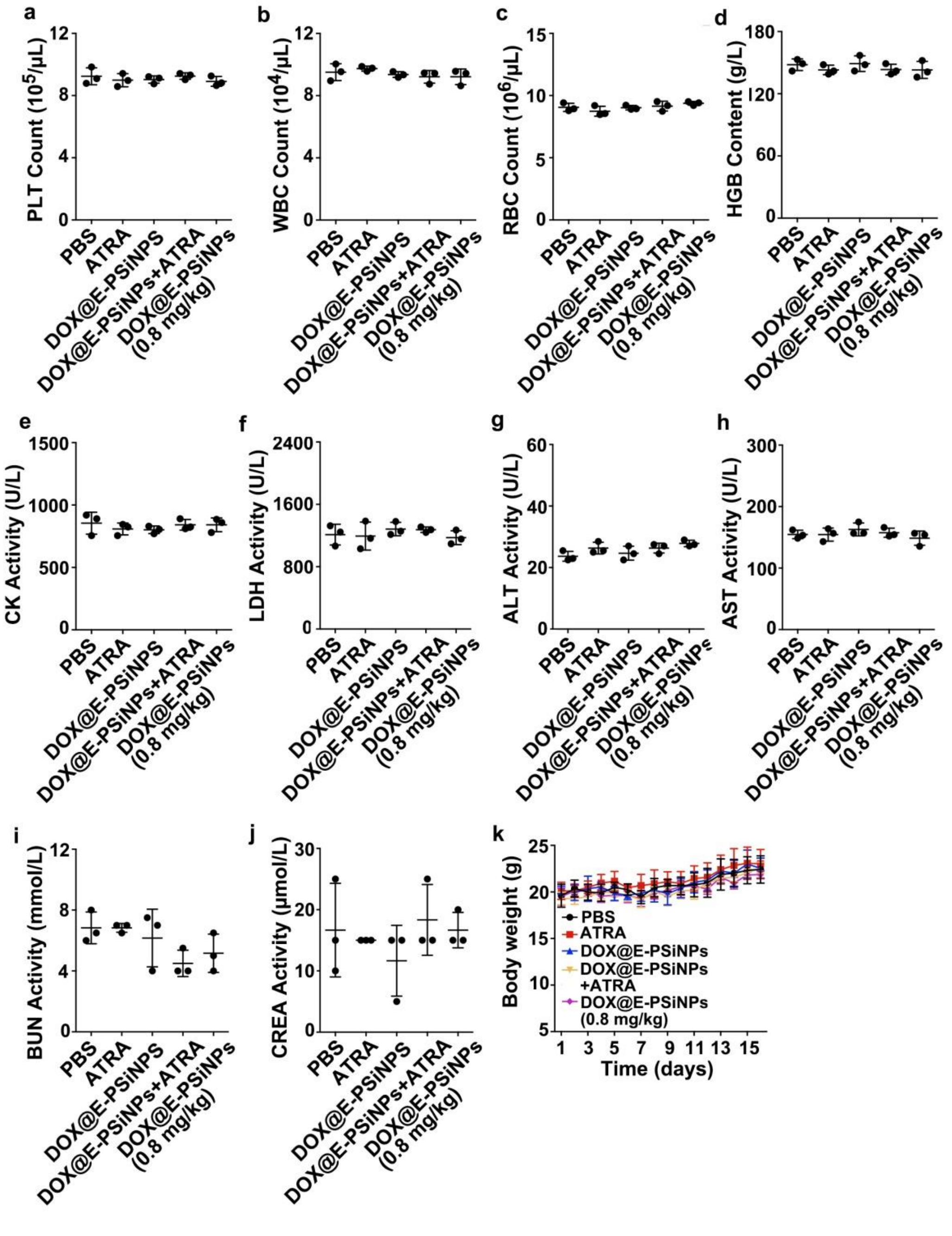

Supplementary Fig. 33. Biosafety evaluation of coinjection of DOX@E-PSiNPs and ATRA in H22 tumor-bearing mice. **a-d** Routine blood test of platelet (PLT, **a**), white blood cell (WBC, **b**), red blood cells (RBC, **c**) and hemoglobin (HGB, **d**) in H22 tumor-bearing mice after intravenous injection of PBS, ATRA, DOX@E-PSiNPs exocytosed from H22 cells or combination of DOX@E-PSiNPs and ATRA at ATRA and DOX dosage of 4 mg/kg and 0.5 mg/kg, respectively, or DOX@E-PSiNPs at

DOX dosage of 0.8 mg/kg every three days for five times. **e-j** Serological analysis of CK (**e**), LDH (**f**), ALT (g), AST (**h**), BUN (**i**) and CREA (**j**) activity after treatment as above. **k** Body weight of H22 tumor-bearing after treatment as above. Data were represented as mean ± SD (n=3). *P<0.05, **P<0.01 (one-way ANOVA with Bonferroni's multiple comparison test). Source data are provided as a Source Data file.

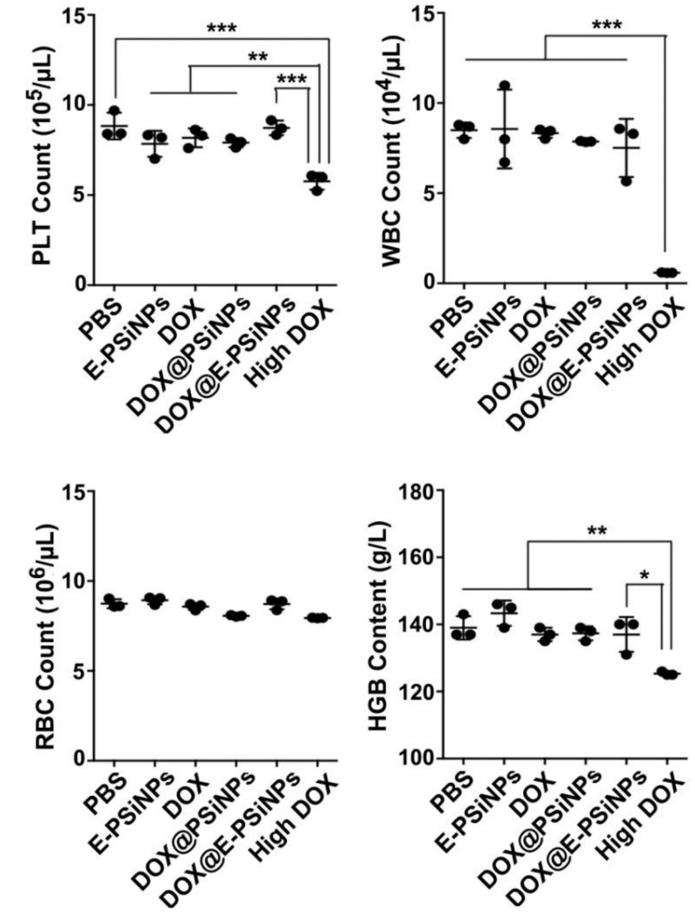

Supplementary Fig. 34. Routine blood test of PLT, WBC, RBC and HGB in orthotopic 4T1 tumor-bearing mice after intravenous injection of PBS, E-PSiNPs, free DOX, DOX@PSiNPs or DOX@E-PSiNPs at DOX dosage of 0.5 mg/kg, or free DOX at 4 mg/kg dosage once every three day for 5 times. Data were represented as mean ± SD (n=3). *P<0.05, **P<0.01, ***P<0.001 (one-way ANOVA with Bonferroni's multiple comparison test). Source data are provided as a Source Data file.

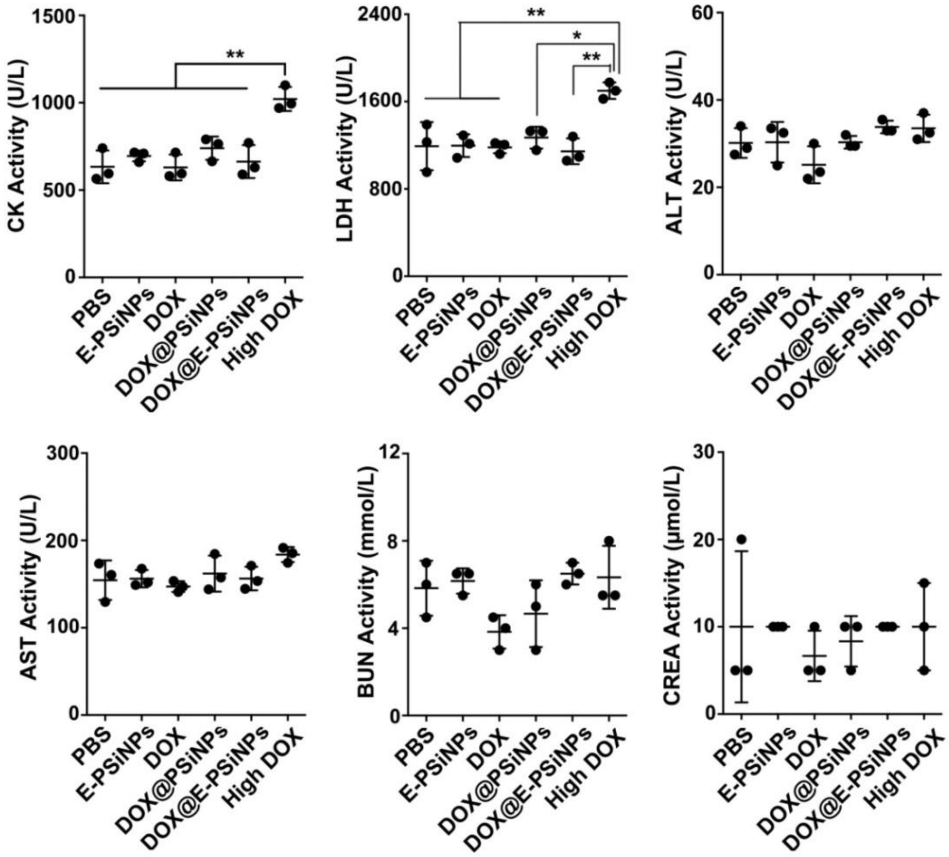

Supplementary Fig. 35. Serological analysis of CK, LDH, ALT, AST, BUN and CREA in orthotopic 4T1 tumor-bearing mice after intravenous injection of PBS, E-PSiNPs, free DOX, DOX@PSiNPs or DOX@E-PSiNPs at DOX dosage of 0.5 mg/kg, or free DOX at 4 mg/kg dosage once every three day for 5 times. Data were represented as mean ± SD (n=3). *P<0.05, **P<0.01 (one-way ANOVA with Bonferroni's multiple comparison test). Source data are provided as a Source Data file.

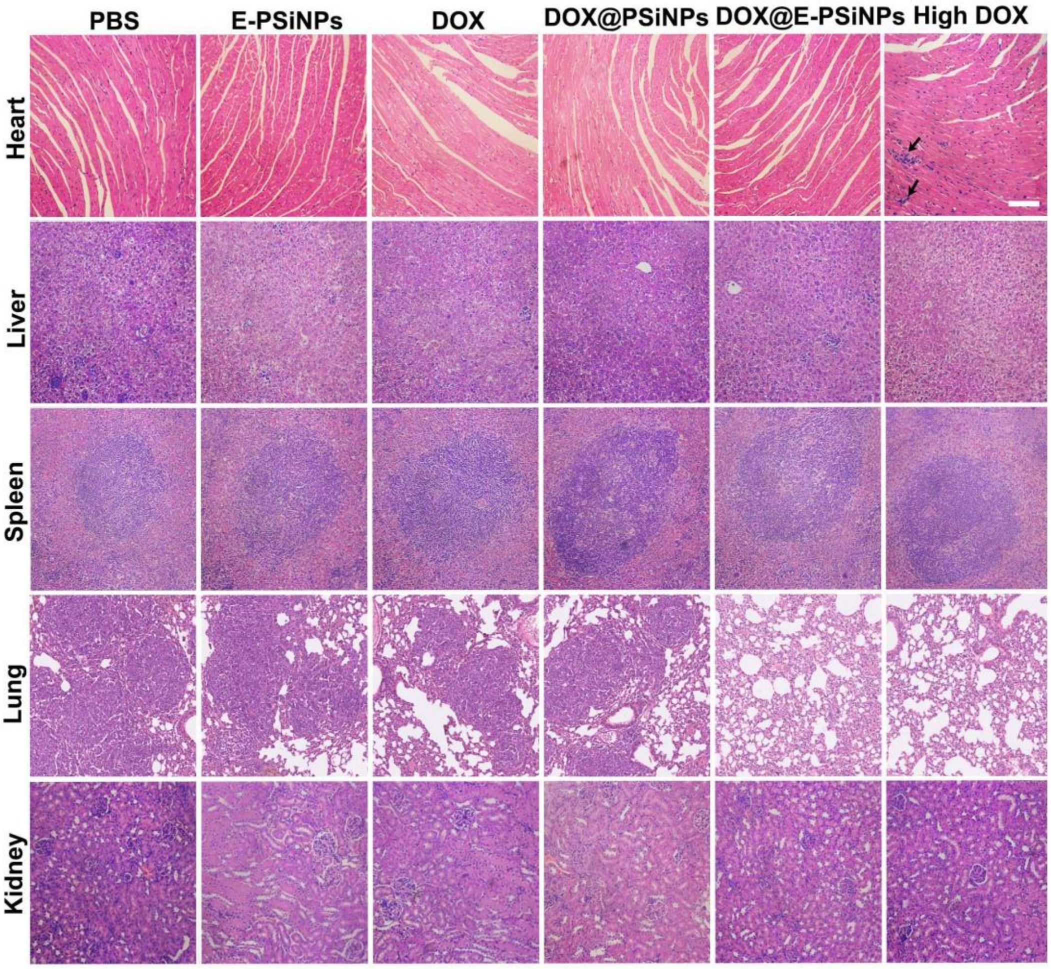

Supplementary Fig. 36. H&E staining of major organs of orthotopic 4T1 tumor-bearing mice after intravenous injection of PBS, E-PSiNPs, free DOX, DOX@PSiNPs or DOX@E-PSiNPs at DOX dosage of 0.5 mg/kg, or free DOX at 4 mg/kg dosage once every three day for 5 times. Black arrows represent neutrophil accumulation. Scale bar: 50 μm.

.

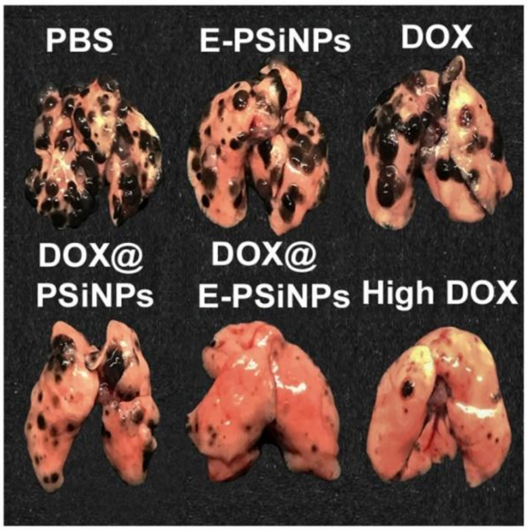

Supplementary Fig. 37. Representative images of lungs of B16-F10 tumor-bearing mice after intravenous administration of different formulations.

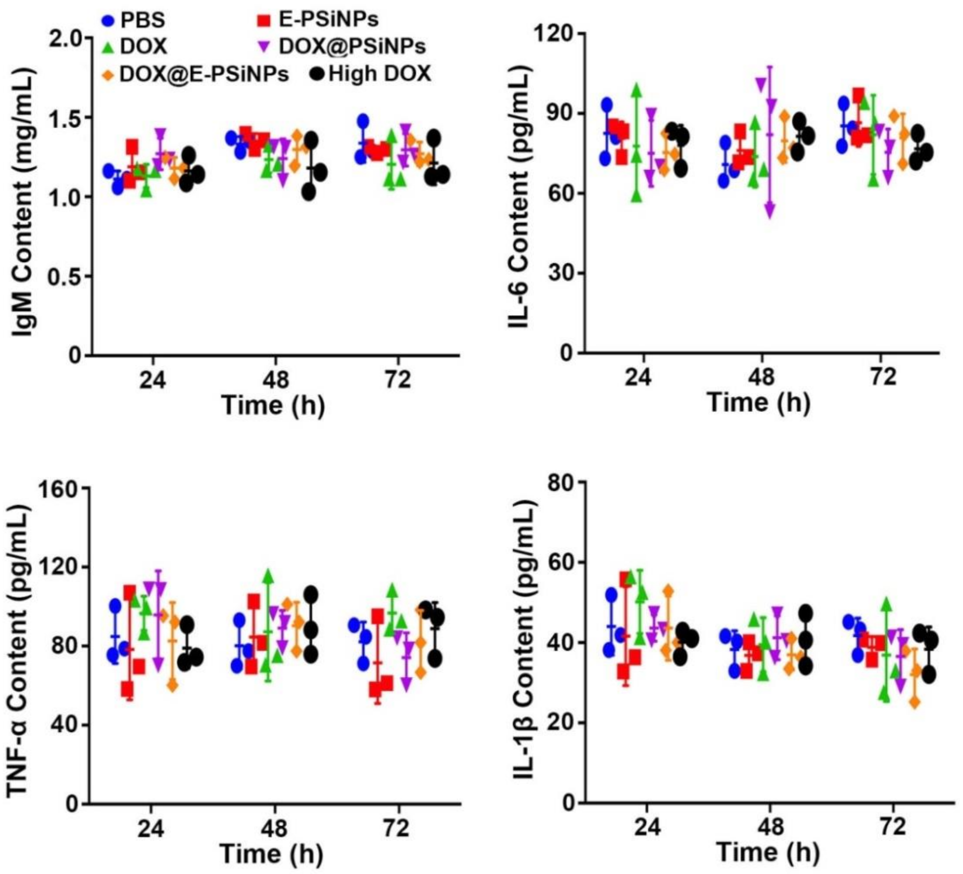

Supplementary Fig. 38. IgM, IL-6, TNF-α and IL-1β contents in serum of C57BL/6 mice at different time intervals after intravenous injection of PBS, E-PSiNPs, free DOX, DOX@PSiNPs or DOX@E-PSiNPs exocytosed from H22 cells at DOX dosage of 0.5 mg/kg, or free DOX at 4 mg/kg dosage. Data were represented as mean ± SD (n=3). Source data are provided as a Source Data file.

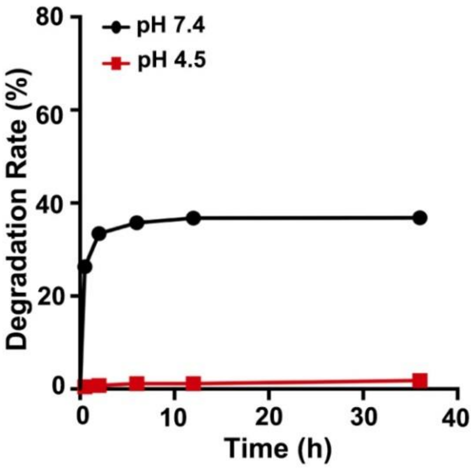

Supplementary Fig. 39. Degradation rate of PSiNPs in PBS at pH 7.4 or 4.5. Data were represented as mean ± SD (n=3). Source data are provided as a Source Data file.
